# Targeting asparagine synthetase triggers ferroptosis and suppresses endometrial cancer growth *via* Atractylenolide II

**DOI:** 10.1515/jtim-2026-0069

**Published:** 2026-08-24

**Authors:** Shuang Tian, Chao Liu, Jie Wu

**Affiliations:** Department of Cell Biology and Genetics, Basic Medical College, Jinzhou Medical University, Jinzhou, Liaoning Province, China; Liaoning Province Key Laboratory of Human Phenome Research, Jinzhou Medical University, Jinzhou, Liaoning Province, China; School of Public Health, Shenyang Medical College, Shenyang, Liaoning Province, China; Liaoning Medical Functional Food Professional Technology Innovation Center, Shenyang Medical College, Shenyang, Liaoning Province, China

## To the editor

Uterine corpus endometrial carcinoma (UCEC) is one of the most commonly gynecological malignant cancer with high incidence and mortality rates, which severely threatens female health and quality of life.^[1]^ Despite of the good prognosis for UCEC patients with early-stage, women diagnosed with advanced stage or recurrent of disease still have poor clinical outcomes, with a 5-year survival rate only about 17%.^[2]^ Therefore, there is the increasing demand of screening out effective biomarkers and potential targeted drugs for clinical diagnosis and treatment of UCEC. Asparagine synthetase (ASNS) mainly located in the cytoplasm of most mammalian cells is a key enzyme involved in amino acid metabolism and highly expressed ASNS in many tumors were associated with poor prognosis.^[3, 4, 5]^ Ferroptosis is characterized by the intracellular iron-dependent lethal accumulation of membrane-localized lipid peroxides and regulated by multiple cellular metabolic events, including metabolism of amino acids, mitochondrial activity, redox homeostasis, iron handling, and lipids and sugars.^[6]^ Extensive studies suggested ferroptosis acted as an innate tumor suppressor factor and participates in the multiple biological processes of UCEC. Thus, targeting ferroptosis signaling pathway is considered as a new challenges and opportunities for endometrial cancer therapy.^[7]^ This study aimed to investigate the impacts of ASNS on endometrial cancer cell ferroptosis and its potential targeted drug.

The mRNA and protein differential expression of ASNS and survival curve were analyzed using University of Alabama at Birmingham Cancer Analytics Portal (UALCAN) database. The univariate and multivariate cox regression analysis were visualized based on XIANTAO database. The protein expression of ASNS in normal and cancer tissues was evaluated by immunohistochemistry (IHC). Cell viability assays, colony formation assays, the levels of intercellular malondialdehyde (MDA), glutathione (GSH), Fe^2+^ and reactive oxygen species (ROS) were detected as functional experiments. Western blot was used to test the expression of NRF2, SLC7A11, VDAC and ACSL4. Molecular docking and molecular dynamics simulation were utilized to explore the binding capability and stability between Atractylenolide II (AT-II) and ASNS. Data were analyzed using GraphPad Prism 8.0. Results are presented as the mean ± standard deviation. One-way analysis of variance, Dunnett's and Tukey's test were used for multiple comparisons in several groups. Unpaired *t* test was employed between the two groups. Wilcoxon test was used to compare IHC scores between the two groups. The correlation of ASNS expression with clinicopathological features was assessed using Fisher test. *P* < 0.05 was considered as statistically significant.

We found the mRNA and protein expression of ASNS were highly expressed in cancer tissues compared with the normal tissues through bioinformatic analysis (Figure 1A). High protein expression of ASNS was confirmed in UCEC tissues by IHC (Figure 1B and Supplementary Figure S1A, *P* = 0.01). The expression level of ASNS was positively related to the histologic grade (Supplementary Figure S1B) and was negatively correlated with overall survival in UCEC patients (Figure 1C). Higher ASNS expression levels were associated with poorer survival outcomes and shorter survival time. The univariate and multivariate analysis data showed that highly expressed ASNS was an independent risk factor for poor prognosis in UCEC patients (Supplementary Figure S1C). These findings suggested that ASNS had good prognostic value and may serve as a novel target for the treatment of UCEC in clinical settings. Therefore, we transiently transfected ASNS siRNAs into AN3CA and Ishikawa cells to identify the biological functions of ASNS in endometrial cancer. The results suggested that siASNS-1 and siASNS-3 were more efficient than siASNS-2 (Supplementary Figure S2A). ASNS knockdown did not affect its enzyme activity (Supplementary Figure S2B), but decreased cell viability, colony formation ability (Supplementary Figure S2C-D) and the levels of GSH, and increased the levels of MDA, Fe^2+^ and intracellular ROS (Figure 1D-1E), accompanied with the decreasing expression of NRF2 and SLC7A11 and the increasing expression of ACSL4 and VDAC (Figure 1F). The findings indicated that ASNS as an oncogene promote proliferation and inhibit ferroptosis in endometrial cancer cells.

**Figure 1:**
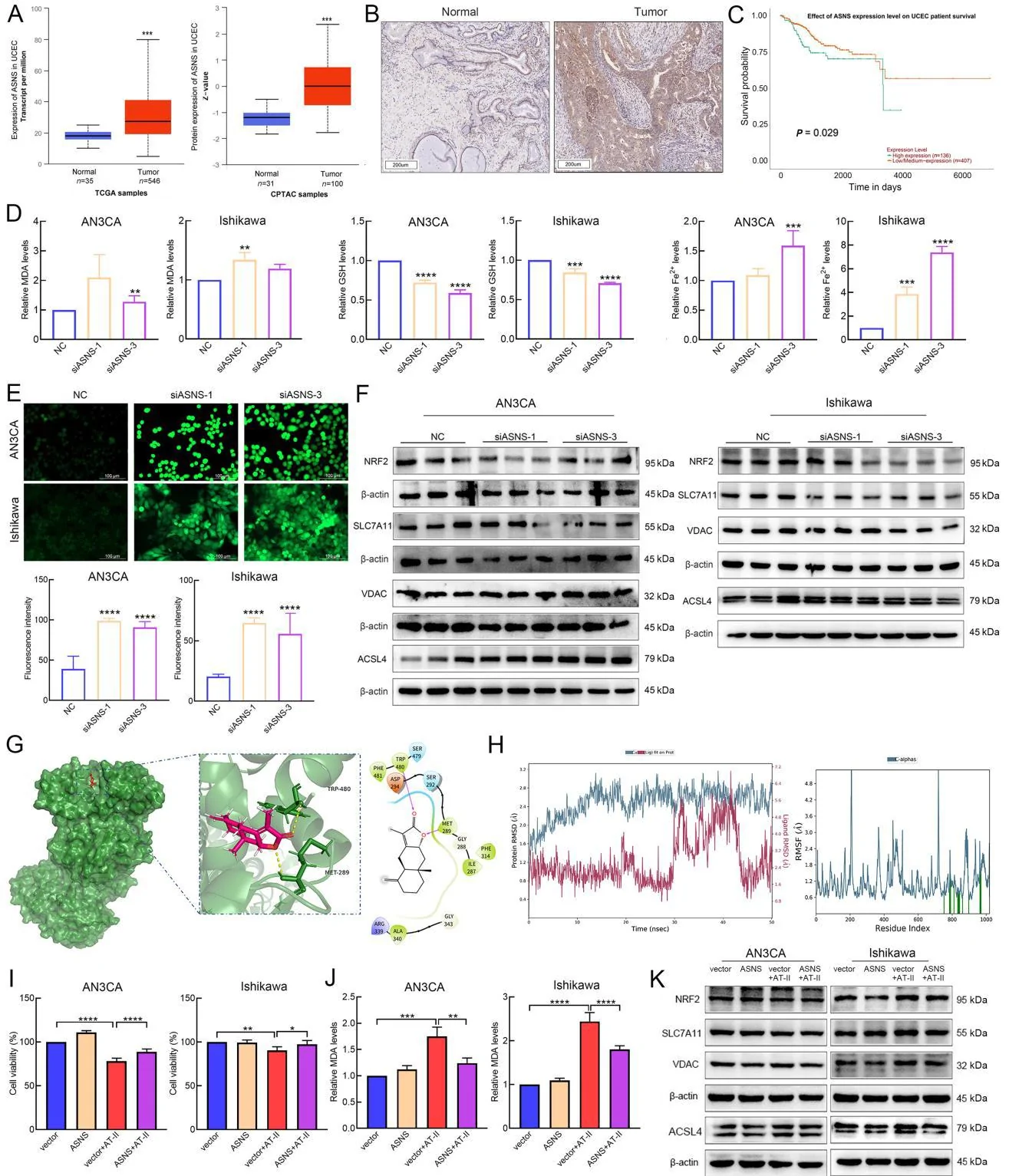
ASNS as a target of AT-II promoted proliferation and inhibited ferroptosis in endometrial cancer. (A) The mRNA and protein expression level of ASNS in UCEC from UALCAN database. (B) Representation of IHC staining images. (C) The relationship between ASNS and overall survival. (D) ASNS knockdown increased MDA production, the consumption of GSH, and Fe^2+^ level. (E) ASNS silencing improved the level of intracellular ROS. (F) Inhibition of ASNS affected the protein expression of NRF2, SLC7A11, VDAC and ACSL4. (G) 3D structure and 2D structure of the compound. (H) RMSD and RMSF map of the ASNS protein-compound complex. (I) ASNS overexpression reversed the AT-II-induced inhibition of cell viability. (J) ASNS overexpression attenuated the AT-II-induced production of MDA. (K) ASNS overexpression affected the protein expression of NRF2, SLC7A11, VDAC and ACSL4. *P < 0.05, **P < 0.01, ***P < 0.001, ****P < 0.0001. ASNS: asparagine synthetase; AT-II: Atractylenolide II; UCEC: uterine corpus endometrial carcinoma; UALCAN: University of Alabama at Birmingham Cancer Analytics Portal; IHC: immunohistochemistry; MDA: malondialdehyde; GSH: glutathione; ROS: reactive oxygen species.

AT-II, the major active compound extracted from traditional Chinese medicine *Baizhu* has remarkable physiological and pharmacological effects, especially anti-cancer activity. Our precious study revealed that AT-II suppressed endometrial cancer cell proliferation and glycolysis by blocking the PADI3-ERK signaling pathway.^[8]^ In this study, we demonstrated that AT-II inhibited cell growth and induced ferroptosis by downregulating the protein expression of NRF2 and SLC7A11, and upregulating the protein expression of ACSL4 and VDAC *in vitro* (Supplementary Figure S3). Additionally, AT-II suppressed xenograft tumor growth by 64% (*P* < 0.01), induced ferroptotic markers, and showed no hepatorenal toxicity (unchanged ALT/AST/CRE/BUN) (Supplementary Figure S4). The roles of AT-II were consistent with those of ASNS knockdown. Therefore, we supposed that ASNS may be a potential target of AT-II in endometrial cancer. Molecular docking and molecular dynamics simulation are pivotal technologies in drug field, which could sufficiently elucidate the dynamic interaction between drug molecules and target proteins.

Molecular docking could predict the interaction patterns and binding affinities between drug molecules and target proteins.^[9]^ Molecular dynamics simulation could analyze the changes of conformational, key residue interactions, and free energy during drug-target binding processes under physiological conditions, thereby overcoming the limitations of molecular docking.^[10]^ Molecular docking showed that AT-II fitted better into the hydrophobic cavity of the pocket, thus confirming the possibility that the compound bound well to the ASNS (Figure 1G). Root Mean Square Deviation (RMSD) analysis revealed that there were partial ups and downs from 30-44 ns, finally reaching equilibrium at around 44 ns. Root Mean Square Fluctuation (RMSF) analysis suggested that there were multiple bindings of the ligand to the protein binding site, indicating that the ligand was able to stay well in the docked pocket during the 50 ns simulation (Figure 1H). We overexpressed ASNS to further confirm that ASNS was the target of AT-II. In the rescue experiment, we found that ASNS overexpression reversed the AT-II-induced inhibition of cell viability (Figure 1I) and the production of MDA (Figure 1J). Western blot results suggested that the AT-II-induced decrease in the expression of NRF2 and SLC7A11 were attenuated in AN3CA cells, the increased in the expression of VDAC and ACSL4 were weakened in AN3CA and Ishikawa cells (Figure 1K). These results indicated that AT-II might trigger ferroptosis and suppress endometrial cancer growth by targeting ASNS. However, there are some limitations in our study, and we will perform relevant experiments to demonstrate the direct binding between ASNS and AT-II in subsequent research.

In conclusion, our study found that ASNS promoted endometrial cancer progression by inhibiting cell ferroptosis signaling pathway and was a potential therapeutic target of AT-II. Therefore, ASNS may serve as a novel therapeutic target for endometrial cancer. The targeted drug AT-II might provide a new perspective for cancer intervention and clinic therapy of human endometrial carcinoma.

## Supplementary Information

Supplementary materials are only available at the official site of the journal (www.intern-med.com).

## Supplementary Material

Supplementary Material Details
