# Supplementary material for: Targeting asparagine synthetase triggers ferroptosis and suppresses endometrial cancer growth via Atractylenolide II
Source: J Transl Int Med. 2026 Aug 24;14(5):842–5. doi: 10.1515/jtim-2026-0069 (PMC13726664; doi:10.1515/jtim-2026-0069)
Supplement: Supplementary file 1 [file jtim-2026-0069_sm.pdf]

Supplementary materials

Targeting asparagine synthetase triggers ferroptosis and suppresses endometrial cancer growth *via* Atractylenolide II

Abstract: Background and Objectives: Asparagine synthetase (ASNS), a key enzyme involved in amino acid metabolism, is upregulated in various types of tumors. However, both the expression and functions of ASNS in endometrial cancer remain unknown. This study aimed to investigate the impacts of ASNS on endometrial cancer cells and its potential targeted drug. **Methods:** ASNS expression in endometrial cancer and the correlation with patient survival time were analyzed based on UALCAN and XIANTAO databases. Immunohistochemistry was used to evaluate the expression of ASNS in normal and cancer tissues. Cell viability assay, colony formation, intercellular MDA, GSH, Fe^{2+} and ROS assay were performed to detect the roles of ASNS. The expression levels of NRF2, SLC7A11, VDAC and ACSL4 were tested by western blot. The binding capability and stability between AT-II and ASNS was analyzed by molecular docking and molecular dynamics simulation. **Results:** ASNS was highly expressed in endometrial cancer tissues and associated with poor prognosis. ASNS knockdown inhibited proliferation and promoted ferroptosis in AN3CA and Ishikawa cells of endometrial cancer by modulating NRF2/VDAC/ACSL4 pathway. AT-II suppressed cell proliferation and induced ferroptosis in vitro and vivo. AT-II had a good binding capability and stability with ASNS and negatively regulated the expression of ASNS. ASNS overexpression attenuated the effects of AT-II on endometrial cancer cells. **Conclusion:** ASNS is a potential biomarker and therapeutic target of endometrial cancer. The targeted drug AT-II might provide a new perspective for cancer intervention and clinic therapy of endometrial cancer.

Key words: ASNS; endometrial cancer; ferroptosis; AT-II

Supplementary material 1-Materials and Methods

Bioinformatics analysis

UALCAN database (http://ualcan.path.uab. edu/) was used for analyzing the mRNA and protein differential expression of ASNS, and survival curve. The univariate and multivariate cox regression analysis was visualized by using XIANTAO database (https://www.xiantaozi.com/).

Tissue microarray and immunohistochemistry (IHC) staining

UCEC tissue microarray (Cat HUteA045PG01) comprising 34 UCEC tissue and 10 paired adjacent normal endometrial specimens, purchased from Shanghai Outdo Biotech Co., Ltd. The tissue microarray slide was incubated with the anti-ASNS primary antibody (1:500, Proteintech) and the secondary antibody. The slide was visualized by DAB solution and hematoxylin counterstain. The expression scores of ASNS were calculated by multiplying the staining intensity and positive rate.

Cell culture and transfection

Human endometrial cancer cell lines AN3CA and Ishikawa were obtained from Procell (Wuhan, China). AN3CA cells were cultured in MEM-modified medium and Ishikawa cells were cultured in DMEM medium. All cells were supplemented with 10% fetal bovine serum, 100 U/mL penicillin, and 100 μg/mL streptomycin. The human ASNS siRNAs and negative control (NC) siRNAs were purchased from JTSBIO (Wuhan, China). The sequences were as follows: siASNS-1: forward, 5′-CCAACUGCUGCUUUGGAUUTT-3′, reverse, 5-′AAUCCAAAGCAGCAGUUGGTT-3′; siASNS-2: forward, 5′-GCUGUAUGUUCAGAAGCUATT-3′, reverse, 5′-UAGCUUCUGA ACAUACAGCTT-3′; siASNS-3: forward, 5′-GCCAUUACUGGAUGCCCAAT T-3′, reverse, 5′-UUGGGCAUCCAGUAAUGGCTT-3′; NC: forward, 5′-UUCUCCGAACGUGUCACGU TT-3′, reverse, 5′-ACGUGACACGUUCGGAGAATT-3′. The human ASNS overexpression plasmid pCMV-ASNS-3FLAG and empty vector pCMV3-CON-3FLAG were purchased from Genechem (Shanghai, China). The cells were transfected using Trans-Mate (JTSBIO, Wuhan, China) following the instructions.

Cell viability assay

Cell Counting Kit-8 (CCK-8) (APE&BIO, Houston, USA) was used to determine cell viability. The AN3CA and Ishikawa cells were seeded in 96-well plates at a density of 5000 cells/well and treated with siRNA or AT-II (Chengdu Must Bio-technology Co., Ltd, Chengdu, China) for 48 h. Subsequently, 10 μl of CCK-8 solution was added to each well and incubated for another 2 h. Then, the optical density value of cells was measured at 450 nm.

Colony formation assay

The AN3CA and Ishikawa cells were seeded in 6-well plates at a density of 3×10^5 cells/well and treated with siRNA for 48 h. Then, the AN3CA and Ishikawa cells were collected and seeded in 3.5cm plates at a density of 500 cells/well. After incubation for 14 d, the cells were fixed with methanol for 10 min and stained with1% crystal violet for 15 min. The colonies were washed with PBS and photographed. Colony numbers were counted using Image J (Version 1.37v).

Detection of Malondialdehyde (MDA), Glutathione (GSH), Fe^{2+} and Glu concentration

The AN3CA and Ishikawa cells were seeded in 6-well plates at a density of 3×10^5 cells/well and treated with siRNA or AT-II for 48 h. The treated cells were collected and lysed. The lysis solution was centrifuged to remove the cell precipitate. According to the instructions, the concentrations of MDA, GSH, Fe^{2+}, and Glu in the supernatant were measured using MDA assay kit (Beyotime, Shanghai, China), GSH, Fe and Glu assay kits (Nanjing Jiancheng Bioengineering Institute, Nanjing, China), respectively. The levels of MDA, GSH, Fe^{2+} and Glu were calculated based on a standard curve and normalized relative to the protein concentrations.

The level of intercellular ROS assay

The AN3CA and Ishikawa cells were seeded in 6-well plates at a density of 3×10^5 cells/well and

treated with siRNA or AT-II for 48 h. Cells were rinsed with PBS and incubated with DCFH-DA for 30 min at 37 ℃. Then, cells were observed under fluorescence microscope and took photos. Image J software (Version 1.37v) was used to determine the intensity of fluorescence.

Western blot

Total protein of cells or tissues extracted by RIPA buffer were separated via 10% SDS-PAGE gels and transferred onto polyvinylidene fluoride membranes (Millipore, USA). Then, the membranes were blocked in 1% bovine serum albumin for 1 h at room temperature and incubated overnight with primary antibodies against the target proteins at 4 ℃. Rabbit ASNS antibody (1:000), rabbit nuclear factor erythroid 2-related factor 2 (NRF2) antibody (1:1000), rabbit acyl-coA synthetase long-chain family member 4 (ACSL4) antibody (1:2000), and rabbit solute carrier family 7 member 11 (SLC7A11) antibody (1:1000) were purchased from Proteintech (Chicago, USA). Rabbit mitochondrion voltage-dependent anion channel (VDAC) antibody (1:1000) was purchased from CST (Boston, USA). Next day, the membranes were incubated with horseradish peroxidase (HRP)-conjugated secondary antibody for 1 h at room temperature. Then, the target proteins were examined using an ECL kit (Beyotime, Shanghai, China). β-actin was used as the loading control. The relative protein expression levels were quantified by Clinx Gel Analysis software (Version 2.6.1.0)

Xenograft tumor assay in nude mouse

Female, 5-6 weeks old BALB/c-nu mice were purchased from Liaoning Changsheng Biotechnology Co., Ltd Limited company. 3×10^6 AN3CA cells were injected into the right back of mice, after that, the mice were divided into control and 75 mg/kg AT-II groups by random. Given drugs by intraperitoneal injection for 14 days, the serums, tumors, liver and kidney tissues were collected and tested. The serums were measured by alanine aminotransferase (ALT), aspartate aminotransferase (AST), blood urea nitrogen (BUN) (solarbio, Beijing, China), and Creatinine (CRE) ((Nanjing Jiancheng Bioengineering Institute, Nanjing, China)) assay kits. Liver and kidney tissues were detected by hematoxylin and eosin (HE) staining. The supernatants obtained from tumors lysed by RIPA buffer were used to determine the levels of MDA, GSH, and Fe^{2+}. Tumor tissues also were used to detect the protein expression of nuclear proliferation-associated antigen ki67 (1:500, abclonal, Wuhan, China) by IHC. All animal experiments have been approved by the Animal Ethics Committee of Jinzhou Medical University and all experimental operating procedures complied with animal ethics requirements (Approval: 240104).

Molecular docking and molecular dynamic simulation

Protein preparations: Download the ASNS protein in PDB format (PDB ID: 6GQ3) from the PDB website (https://www.rcsb.org/).[1] The protein was prepared by importing it into Schrodinger 2021 and pre-processed in the Prepwizard module. The hydrogen bonding network was optimized by sampling the terminal X-angle 180° flips of the Asn, Gln, and His residues and by sampling the hydrogen above the hydroxyl and thiol groups to optimize the hydrogen bonding network. After optimization of the hydrogen bonds, the protein structure was minimized using Impact's Impref

module and the OPLS-2005 force field, allowing the relaxation of the entire structural system. In the protein minimization scheme, all-atom or pure hydrogen atoms are included, while the conditional criterion for termination is based on the root-mean-square deviation of the heavy atoms with respect to their initial position. At the same time, all water molecules are removed under the premise that the hydrogen bonding is optimized and the necessary water molecules are retained in the minimization phase.

Molecular docking: The molecular docking studies were carried out using the Maestro program and the ray structures of the ASNS proteins with a resolution of 1.85 A were selected for the receptor for the molecular docking experiments, the ligands were prepared in Schrodinger's Ligprep module and the various stereoisomers of the ligands, the reciprocal isomers and the protonated and ionized states of the ligands were generated using an ion generator at pH=7.4. We used the Glide module to dock the prepared receptors to the ligands, selecting the XP docking accuracy and adding the molecular protonation state to the scoring. A threshold of 2.5 kcal/mol was set for the ring conformation and the input conformation was normalized to select the OPLS3 stance for docking.

Molecular dynamic simulation: Molecular dynamics simulations were performed using the Schrodinger LLC package.[2] The selected complexes were subjected to kinetic calculations in the Desmond module, using a periodic boundary approach to the simulation. The kinetic simulations were carried out at a temperature of 300 K for 100 ns with a time interval of 2 fs. The coordinate trace files were saved at 10 ps intervals.

REFERENCES

1. Zhu W, Radadiya A, Bisson C, et al. High-resolution crystal structure of human asparagine synthetase enables analysis of inhibitor binding and selectivity. Commun Biol. 2019;2:345.
2. Bowers KJ, Chow E, Xu HF, et al. Scalable algorithms for molecular dynamics simulations on commodity clusters in ACM/IEEE SC 2006 Conference. 2006;43.

Supplementary material 2-Supplementary Figures

A

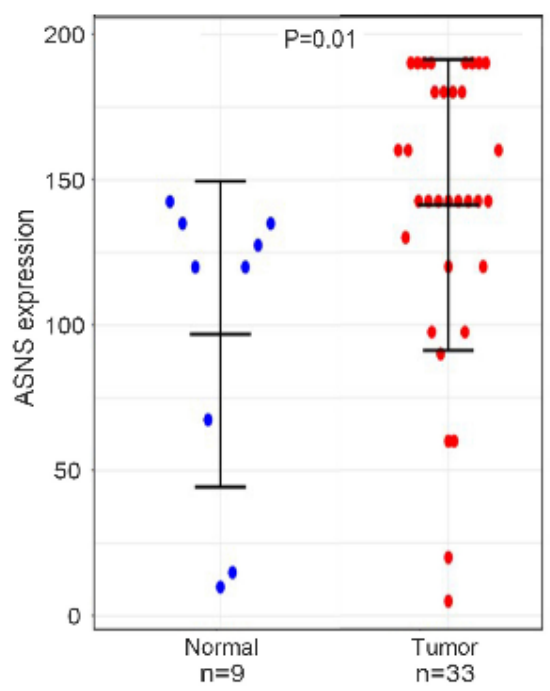

B

Correlation between ASNS expression and clinicopathological characteristics

Characteristics	ASNS expression		P value	R value
	Low	High		
Age			0.721	0.111
≤60	11	7		
>60	7	7		
Grade			0.001	0.59
I/II	17	6		
III	1	9		
Tumor size			1	-0.018
≤4cm	10	9		
>4cm	6	5		

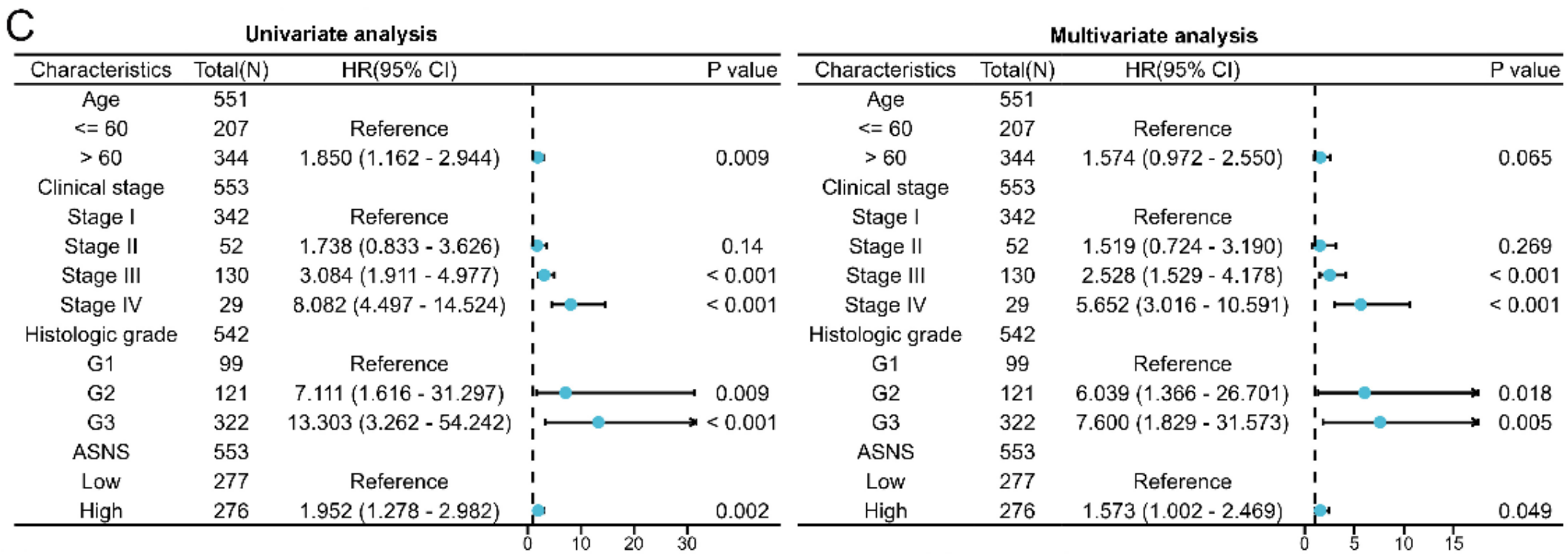

Supplementary Figure S1: (A) Statistical analysis of IHC. (B) The correlation between ASNS and clinicopathological characteristics. (C) The univariate and multivariate cox regression analysis. ASNS: asparagine synthetase; IHC: immunohistochemistry.

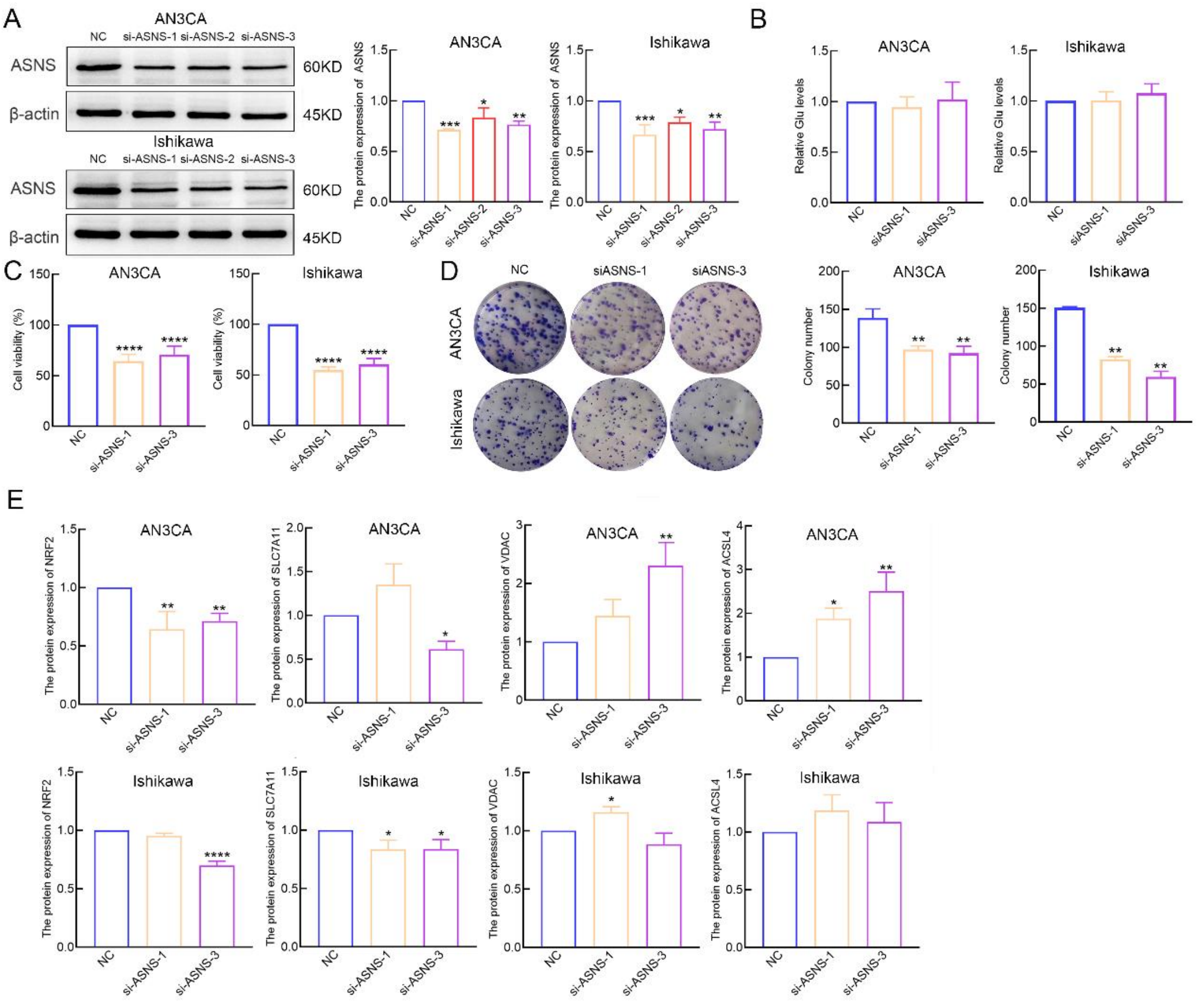

Supplementary Figure S2: (A) Western blot analysis was used to verify the efficacy of ASNS knockdown. (B) The effect of ASNS knockdown on the production of Glu. (C) ASNS positively regulated cell viability. (D) Downregulation of ASNS decreased the colony formation ability of AN3CA and Ishikawa cells. (E) Statistical analysis of the protein expression of NRF2, SLC7A11, VDAC and ACSL4. ASNS: asparagine synthetase.

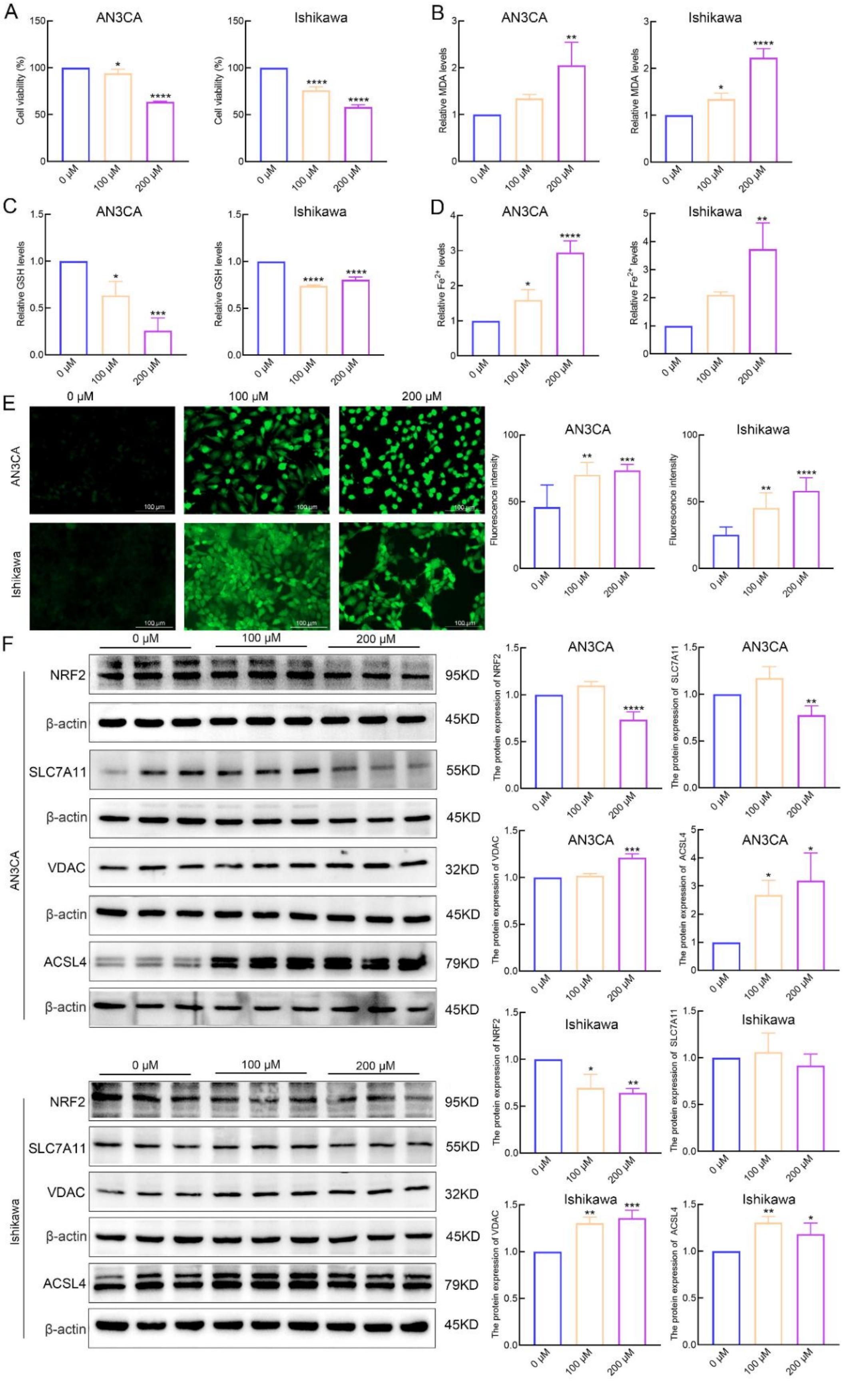

Supplementary Figure S3: (A) AT-II inhibited cell viability. (B-D) AT-II increased MDA production (B), the consumption of GSH (C), and Fe^{2+} level (D) in AN3CA and Ishikawa cells. (E) AT-II improved the level of

intracellular ROS of AN3CA and Ishikawa cells in dose manner. (F) AT-II affected the protein expression of NRF2, SLC7A11, VDAC and ACSL4. AT-II: Atractylenolide II.

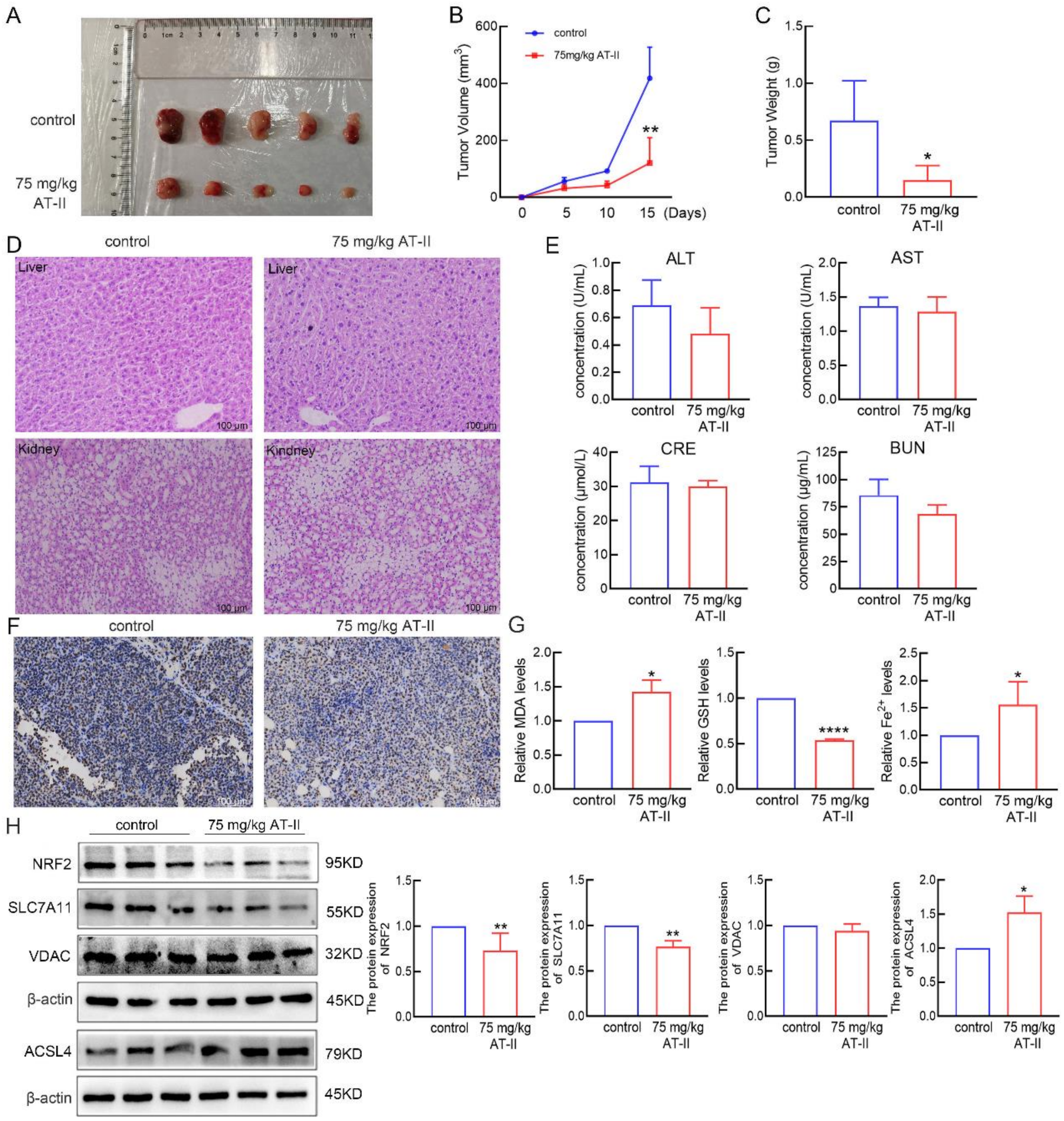

Supplementary Figure S4: (A) The representation of tumor tissues. (B) AT-II inhibited xenograft tumor volume. (C) AT-II reduced xenograft tumor weight. (D) The morphology changes of liver and kidney tissues were detected by HE staining. (E) The concentrations of ALT, AST, CRE, and BUN in serums were measured by assay kits. (F) The expression of ki67 in tumor tissues was tested by IHC. (G) AT-II increased the levels of MDA (left), the consumption of GSH (middle), and the concentrations of Fe^{2+} (right). (H) AT-II affected the protein expression of NRF2, SLC7A11, VDAC and ACSL4. AT-II: Atractylenolide II.
